# Prevalence, incidence, and Disability-Adjusted Life Years of self-harm and suicide mortality in the Middle East and North Africa: a sex-specific study based on Global Burden of Disease

**DOI:** 10.3389/fpsyt.2025.1529941

**Published:** 2025-03-21

**Authors:** Moien AB Khan, Sohrab Amiri

**Affiliations:** ^1^ Health and Wellness Research Group, Department of Family Medicine, College of Medicine and Health Sciences, United Arab Emirates University, Al Ain, United Arab Emirates; ^2^ Spiritual Health Research Center, Lifestyle Institute, Baqiyatallah University of Medical Sciences, Tehran, Iran

**Keywords:** prevalence, incidence, self-harm, suicide mortality, global burden of disease, Middle East and North Africa

## Abstract

**Objectives:**

The study examined the prevalence, incidence, and Disability-Adjusted Life Years (DALYs) associated with self-harm across countries in the Middle East and North Africa, while also analyzing suicide mortality. It aims to explore the variations in self-harm and suicide mortality by sex and assess trends in these phenomena from 1990 to 2021.

**Methods:**

Global Burden of Disease 2021 data sources were used in this study. Estimates for all-age counts and age-standardized prevalence rates (per 100,000) were determined for prevalence, incidence, Disability-Adjusted Life Years (DALYs), and suicide mortality. These disease burden indicators were analyzed across the period from 1990 to 2021, and the results were further stratified by sex and location. Additionally, the percentage change observed between 1990 and 2021 was documented. A 95% uncertainty interval was used for each estimate reported.

**Results:**

The age-standardized prevalence of self-harm in the MENA region was 111.82 per 100,000 in 1990, decreasing to 105.84 by 2021. The global age-standardized prevalence rate of self-harm is 182.24 per 100,000 in 2021. Throughout this period, the self-harm rates in the MENA region remained lower than the global average. In 2021, approximately 621,509 individuals in the region were reported to engage in self-harm. In the same year, the age-standardized suicide mortality rate in MENA stood at 3.43 per 100,000, with an estimated total of over 21,000 suicide deaths. The age-standardized DALYs rate of self-harm in MENA was 246.03 per 100,000 in 1990 and decrease to 177.44 per 100,000 in 2021. The gender disparity in 2021 revealed higher self-harm rates among females than males, at 112.57 vs. 99.67 per 100,000, respectively. In contrast, suicide mortality rates were higher in males than females, recorded at 4.83 vs. 1.92 per 100,000.

**Conclusions:**

Although the rates of suicide mortality and self-harm have declined, the overall number of cases has risen alongside population growth. This highlights the necessity for more comprehensive efforts in mental health care, including screening, prevention, treatment, and the accurate identification of risk factors.

## Introduction

Self-harm and suicide are major health and societal issues in the world, and have a great burden on health, especially in low- and middle-income countries (1). Self-harm is defined as “when somebody injures or harms themselves to cope with or express extreme emotional distress and internal turmoil. They do not generally intend to kill themselves, but the results can be fatal” (2). Suicide is defined as “the act of intentionally carrying out an action to kill oneself” (2).

Based on the estimates made during recent decades, age-standardized incidence rates of self-harm are 62.48 per 100,000 and the rate of DALY rates of self-harm were 424.7 per 100,000 (3). The burden of self-harm varies with age and sex (3). The ratio of self-harm in young women to that in men is reported to be 2.6 to 1 (4). In the most recent report published by the Institute for Health Metrics and Evaluation (IHME), the prevalence of self-harm was 8.4 million in females and 7.07 million in males (5). The World Health Organization (WHO) estimates that more than 700,000 people die by suicide every year, of which 77% live in low- and middle-income countries (6). Among the causes of death, 1.3% of deaths are caused by suicide; according to a report published in 2019, suicide was the 17th most common cause of death worldwide (7), and suicide is more prevalent in men than in women (8, 9). One of the super regions of the world that has faced many challenges in recent decades and has affected mental health is MENA (10).

North Africa and the Middle East, including 21 countries, have a range of sociodemographic similarities. However, there are also differences in other dimensions, and these differences exist in health systems (11). Over the past few decades, this super region has witnessed an improvement in morbidity and mortality due to improvements in health care, health education, and socioeconomic development (11). Several factors can affect mental health, especially self-harm and suicide in MENA. First, as mentioned, the highest rates of self-harm and suicide occur in low- and middle-income countries (6). In this regard, a classification of 21 countries in MENA shows that almost two-thirds of these countries are low- and middle-income countries (11). Second, conflict, war, and its consequences, i.e., displacement and migration, are important factors in mental health (12–15). In this respect, MENA has been the center of conflict and war during the last few decades (16). These wars and conflicts have led to injuries, trauma, human crises, destruction of health infrastructure, displacement, and increase in morbidity and mortality (17). Third, demographic changes in MENA are among the most important health issues affecting all aspects of health. In the past, we have seen a trend of population growth in this super region, which led to an increase in the population and, as a result, the demand for healthcare and services; however, in recent years, a population change has been observed with a decrease in the fertility rate (18, 19).

Self-harm and suicide have negative effects on society’s health and impose a significant burden on health and the economic system. The region has faced decades of conflict, war, and displacement, all of which have significantly affected its mental health. Simultaneously, the shared history and cultural similarities among countries in the area provide a valuable context for understanding these mental health challenges. Examining suicidal behaviors is especially crucial, as the region encounters distinct sociopolitical pressures such as ongoing conflict, migration issues, and scarce mental health resources. These factors play a critical role in shaping both the prevalence of self-harm and suicide as well as the patterns of how they are reported. Based on this, this study aimed to investigate the prevalence, incidence, and Disability-Adjusted Life Years (DALYs) caused by self-harm in MENA countries, as well as suicide mortality. Sex differences in self-harm and suicide mortality, as well as investigating the trends of self-harm and suicide from 1990 to 2021, are among the goals of this study.

## Methods

### Data source

This study was based on the Global Burden of Disease 2021 (20). The analysis of disease burden indicators included prevalence, incidence, Disability-Adjusted Life Years (DALYs), Years Lived with Disability (YLDs), Years of Life Lost (YLLs), and mortality data spanning 371 diseases and injuries. It also incorporates estimates of healthy life expectancy (HALE). These metrics are categorized by sex, age groups, and 204 countries and territories, with subnational data provided for 21 of these countries (20). The GBD 2021 analysis utilized 100,983 data sources, including 19,189 newly added specifically for DALYs. Furthermore, 12 new causes were introduced, accompanied by several major methodological improvements (20). The estimates covered the Middle East and North Africa region, with additional details about GBD 2021 available from other sources (20).

### Case definitions

Self-harm in the GBD 2021 is “deliberate bodily damage inflicted on oneself, resulting in death or injury. ICD-9: E950-E959; ICD-10: X60-X64.9, X66-X84.9, Y87.0” (20, 21). Contains two subclasses Self-harm by firearm “Death or disability inflicted by the intentional use of a firearm on oneself. ICD-9: E955-E955.9; ICD-10: X72-X74.9” (20, 21). Self-harm by other specified means is defined as “death or occurrence of deliberate bodily damage inflicted on oneself resulting in death using self-poisoning, medication overdose, transport, falling from height, hanging or strangulation, or other mechanisms not including firearms. ICD9: E950-E954, E956-E959; ICD10: X60-X64.9, X66-X67.9, X69-X71.9, X75-X75.9, X77-X84.9, Y87.0” (20, 21). This study incorporated suicide data from various sources, including vital registration (VR) records, survey results, verbal autopsy (VA) data, and surveillance systems, with further details available in other referenced materials (22).

### Estimation framework

Years Lived with Disability (YLDs) were calculated “with a microsimulation process that used estimated age-sex-location-year-specific prevalent counts of non-fatal disease sequelae (consequences of a disease or injury) for each cause and disability weights for each sequela as the input estimates at varying levels of severity by an appropriate disability weight” (20). YLLs calculated as “the product of estimated age-sex location-year-specific deaths and the standard life expectancy at the age death occurred for a given cause” (20). DALYs were calculated as the sum of YLDs and YLLs (20).

### Statistical analysis

Estimates for all-age counts and age-standardized rates (per 100,000) were computed for prevalence, incidence, Disability-Adjusted Life Years (DALYs), and suicide mortality. The age-standardized rate is “a weighted average of the age-specific rates, where the weights are the proportions of a standard population in the corresponding age groups” (23). Disease burden indicators were analyzed for the period spanning 1990 to 2021, broken down by sex and location. In addition, the percentage change over the years was recorded. A 95% uncertainty interval was used for each estimate. More details about the data, data processing, and modeling are provided elsewhere and are related to GBD 2021 (20).

GBD 2021 complies with the Guidelines for Accurate and Transparent Health Estimates Reporting (GATHER) (24).

## Results

### Prevalence of self-harm in MENA from 1990 to 2021

**Figure 1:**
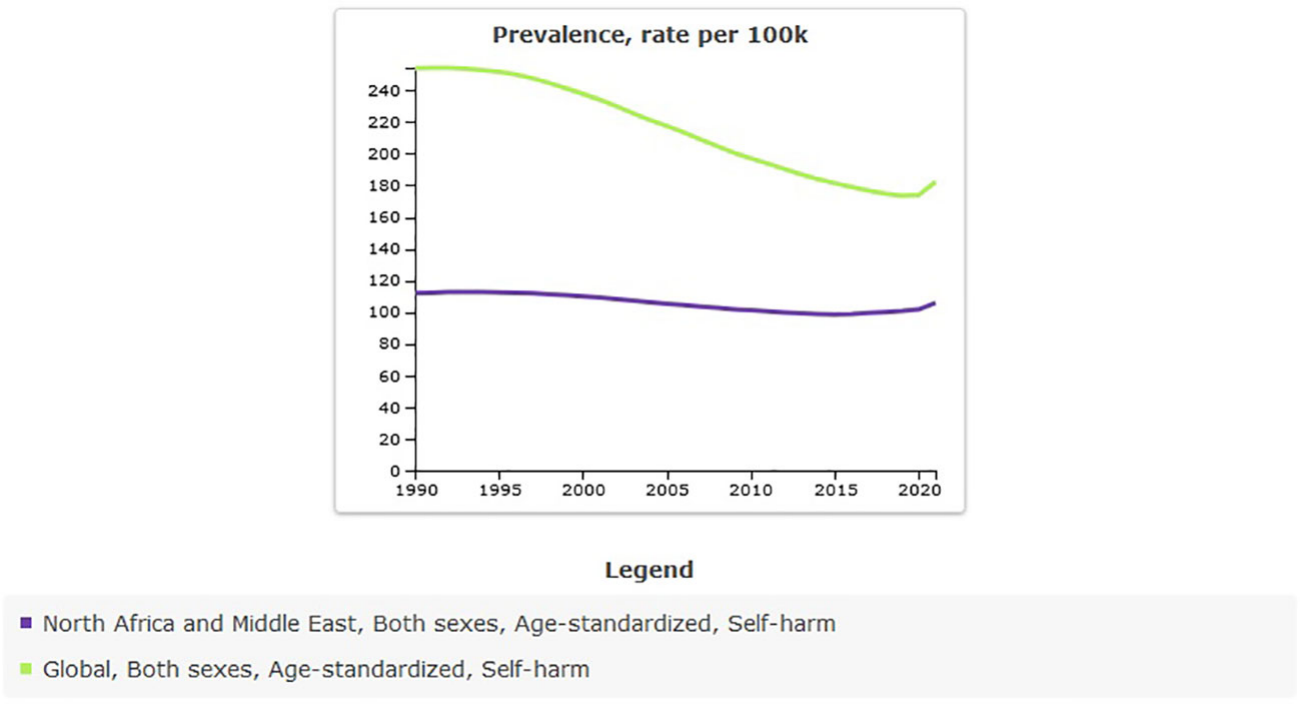
Trend in age-standardized prevalence rate of self-harm in MENA, 1990–2021.

**Figure 2:**
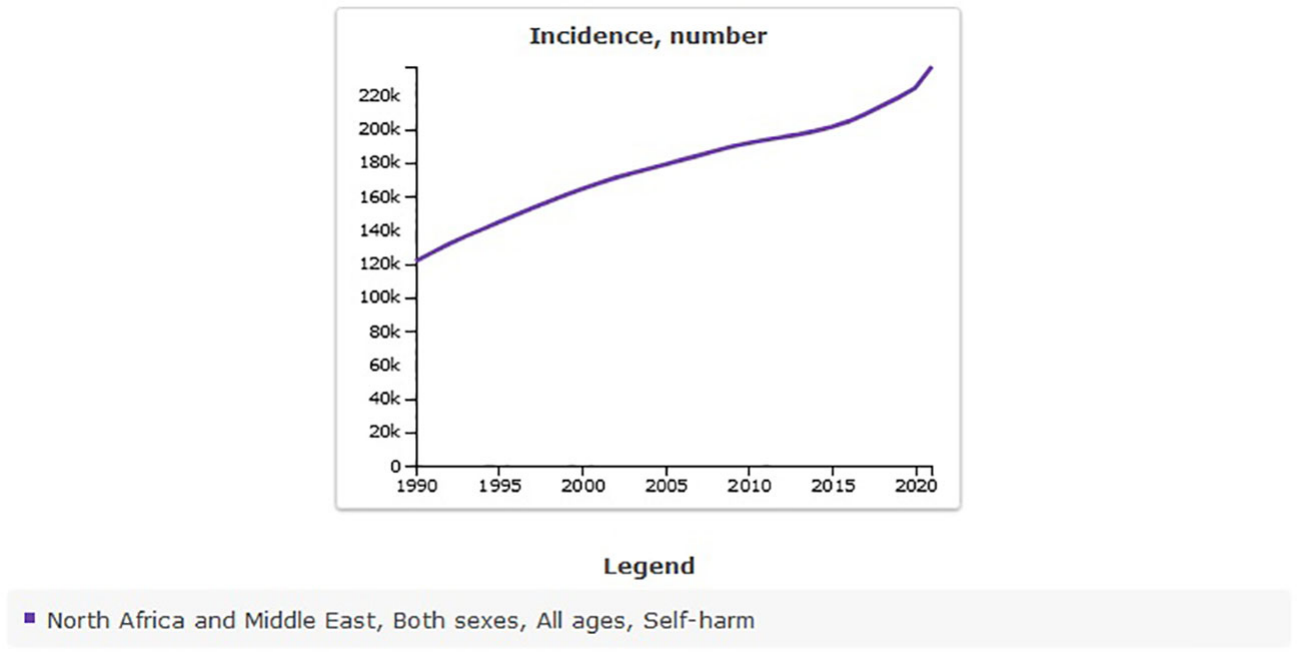
Age-standardized prevalence rate of self-harm globally, 1990–2021.

The age-standardized prevalence rate of self-harm in MENA was 111.82 [95% UI 96.54 to 131.55] per 100,000 in 1990, and this rate decreased to 105.84 [95% UI 90.98 to 125.05] per 100,000 in 2021 [**Table 1**]. The percentage change from 1990 to 2021 showed a negative trend of -5% [95% UI −8 to −4]. Global age-standardized prevalence rate of self-harm was 182.24 per 100,000 in 2021. Compared with the global population, self-harm was less prevalent in MENA from 1990 to 2021 (**Figure 1**). The prevalence of self-harm in MENA countries is lower than that in most other countries (**Figure 2**).

**Table 1:** All-ages counts and age-standardized prevalence rate (per 100,000) of self-harm in MENA, 1990–2021.

Location	1990			2021		
	Value	Lower	Upper	Value	Lower	Upper
Age-standardized prevalence Rate (Per 100,000)						
North Africa and Middle East	111.82	96.54	131.55	105.84	90.98	125.05
Afghanistan	111.88	97.34	129.75	104.34	91.01	120.48
Algeria	134.81	114.37	160.77	103.58	88.6	123.32
Bahrain	95.35	83.03	111.58	119.22	101.53	143.03
Egypt	81.73	71.01	95.66	88.39	76.52	103.49
Iran	173.92	146.13	208.75	131.2	110.55	156.67
Iraq	109.05	94.87	127.56	118.2	102.07	138.7
Jordan	82.1	71.97	94.97	76.66	66.65	90.16
Kuwait	73.08	63.79	85.76	88.8	76.36	104.62
Lebanon	102.26	87.91	121.35	107.58	91.66	128.63
Libya	102.34	87.52	120.18	114	97.78	134.77
Morocco	144.02	123.75	168.26	134.51	115.37	156.85
Oman	73.49	64.03	85.59	82.61	70.8	98.38
Palestine	70.76	61.81	82.26	83.91	72.69	99.07
Qatar	102.74	90.11	120.04	96.75	83.77	113.44
Saudi Arabia	53.52	47.02	62	95.17	82.38	110.53
Sudan	130.66	111.66	154.11	126.57	107.84	150.33
Syrian Arab Republic	60.21	52.49	69.83	78.94	68.72	92.79
Tunisia	88.23	76.62	103.65	96.41	82.67	114.15
Türkiye	95.55	83.68	110.01	89.94	77.67	106.44
United Arab Emirates	97.25	85.72	112.19	106.14	92.21	123.58
Yemen	100.3	87.38	116.27	111.62	96.54	130.96
All-ages counts estimates						
North Africa and Middle East	267,304.61	228,778.94	318,669.66	621,509.77	531,046.77	739,915.39
Afghanistan	7,909.47	6,927.91	9,146.10	18,858.01	16,236.62	22,177.90
Algeria	23,247.34	19,572.84	28,056.27	44,675.15	38,051.79	53,409.93
Bahrain	390.11	332.87	464.21	2,011.37	1,701.57	2,437.11
Egypt	33,490.41	28,736.89	39,470.22	77,620.71	66,617.93	91,756.83
Iran	65,834.87	55,038.75	79,967.61	122,781.76	103,040.92	147,165.99
Iraq	12,522.28	10,752.90	14,795.54	40,557.01	34,775.12	48,026.69
Jordan	1,833.36	1,579.83	2,158.45	8,325.41	7,153.27	9,888.32
Kuwait	1,014.58	867.24	1,216.92	4,673.31	3,991.38	5,593.46
Lebanon	2,595.39	2,234.40	3,091.57	6,536.60	5,556.50	7,852.53
Libya	2,832.41	2,414.23	3,359.13	8,334.35	7,105.74	9,909.74
Morocco	27,072.54	23,075.18	32,003.19	51,204.98	43,881.75	59,844.69
Oman	1,009.97	863.55	1,206.67	3,709.37	3,106.59	4,514.89
Palestine	865.5	746.67	1,018.27	3,281.85	2,807.36	3,917.45
Qatar	390.9	333.87	466.92	3,080.66	2,612.67	3,696.63
Saudi Arabia	5,748.03	4,962.64	6,776.36	37,008.59	31,506.94	43,731.43
Sudan	17,340.03	14,735.78	20,735.97	40,074.99	33,761.26	48,175.35
Syrian Arab Republic	4,692.16	4,039.52	5,512.92	11,257.51	9,819.87	13,190.99
Tunisia	5,681.90	4,890.12	6,704.71	12,970.76	11,110.96	15,340.59
Türkiye	43,702.56	38,003.76	50,617.99	85,080.45	73,573.38	100,502.85
United Arab Emirates	1,431.58	1,225.22	1,713.30	13,057.58	11,130.04	15,351.78
Yemen	7,553.00	6,536.83	8,839.71	25,829.63	22,042.46	30,732.54

The all-age count estimates that the prevalence of self-harm in MENA was 267,304 [95% UI 228,778 to 318,669] in 1990, and this rate increased to 621,509 [95% UI 531,046 to 739,915] in 2021. The percentage change from 1990 to 2021 showed a sharp increase of 133% [95% UI 124 to 140]. This shows that, despite the decrease in the age-standardized prevalence rate, the number of self-harms has increased. An increase in population leads to an increase in self-harm counts.

### Incidence of self-harm in MENA from 1990 to2021

**Figure 3:**
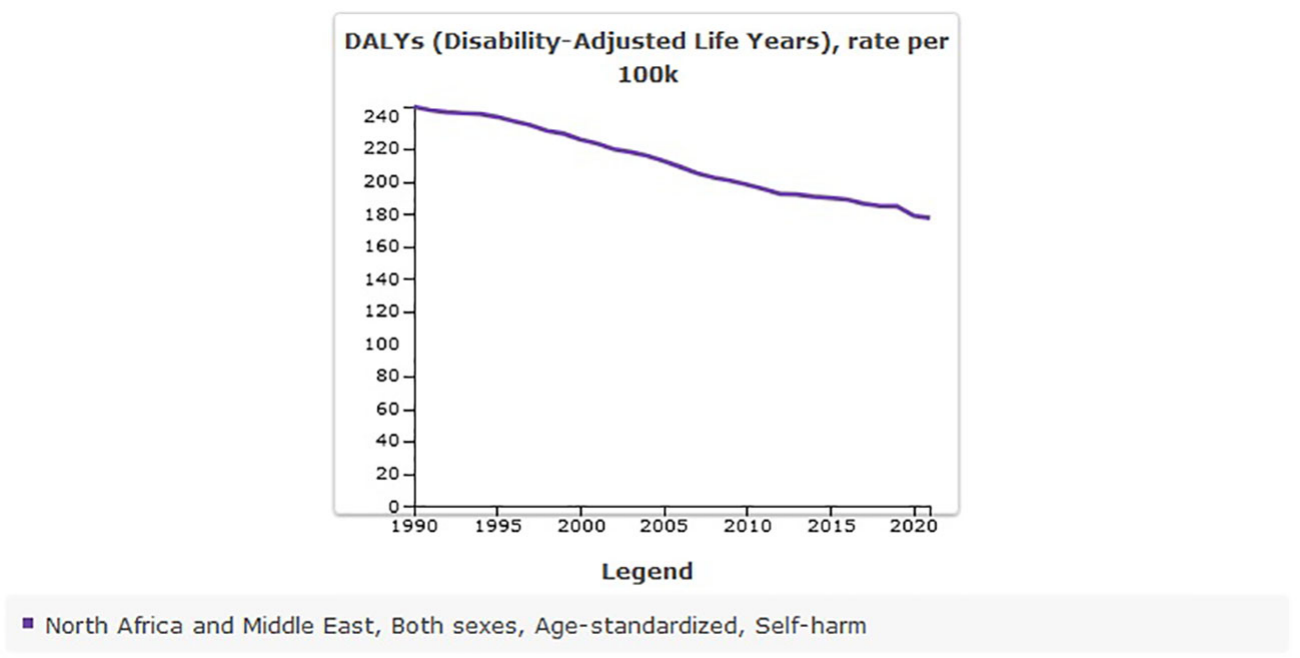
Trend in age-standardized incidence rate of self-harm in MENA, 1990–2021.

The age-standardized incidence rate of self-harm in MENA was 36.08 [95% UI 30.83 to 41.49] per 100,000 in 1990, and there was a very slight increase to 36.52 [95% UI 30.2to 43.85] per 100,000 in 2021 (**Table 2**). The percentage change from 1990 to 2021 shows a positive trend of 1% [95% UI −4 to 7]. The incidence rate of self-harm from 1990 to 2021 showed an almost constant trend with a slight fluctuation (**Figure 3**).

**Table 2:** All-ages counts and age-standardized incidence rate (Per 100,000) of self-harm in MENA, 1990–2021.

Location	1990			2021		
	Value	Lower	Upper	Value	Lower	Upper
Age-standardized incidence Rate (Per 100,000)						
North Africa and Middle East	36.08	30.83	41.49	36.52	30.2	43.85
Afghanistan	38.56	33.4	43.87	34.73	29.3	40.84
Algeria	43.21	37.13	49.87	36.11	30.1	43.04
Bahrain	31.28	26.99	35.68	41.91	35.64	48.53
Egypt	25.93	22.23	29.62	30.44	25.05	36.7
Iran (Islamic Republic of)	57.34	46.67	69.95	46.01	36.07	57.53
Iraq	35.53	30.73	40.4	41.49	34.91	48.4
Jordan	27.4	23.96	30.72	27.31	22.05	33.67
Kuwait	25.27	21.8	29.01	32.66	26.79	39.56
Lebanon	34.43	29.42	39.5	38.95	32.14	46.65
Libya	33.45	28.82	38.3	40.08	33.37	47.63
Morocco	46.03	39.4	52.71	46.12	38.7	54.53
Oman	23.4	19.99	26.98	28.77	23.31	35.48
Palestine	22.59	19.24	26.29	28.82	23.56	35.24
Qatar	33.91	29.26	38.32	34.59	28.88	41.06
Saudi Arabia	16.71	13.94	19.88	33.62	27.62	39.87
Sudan	41.41	35.42	47.69	41.57	33.97	49.88
Syrian Arab Republic	18.95	16.06	22.11	28.01	22.67	34.33
Tunisia	28.96	24.87	33.2	34.3	28.03	40.99
Türkiye	29.84	25.99	33.42	31.9	26.78	37.63
United Arab Emirates	31.1	27.07	35.29	35.84	29.61	42.69
Yemen	32.49	27.39	37.68	36.61	30.26	43.86
All-ages counts estimates						
North Africa and Middle East	121,495.91	101,913.55	142,793.80	237,245.31	194,870.01	284,896.08
Afghanistan	3,231.66	2,766.92	3,787.31	9,858.07	8,072.91	12,041.47
Algeria	11,179.92	9,396.39	13,094.70	15,903.78	13,345.60	18,851.03
Bahrain	176.54	150.83	202.14	743.94	639.03	857.82
Egypt	14,442.24	12,178.87	16,881.42	32,092.19	26,104.04	38,977.36
Iran (Islamic Republic of)	32,083.13	25,531.07	40,402.50	40,908.57	32,066.60	50,434.56
Iraq	6,166.07	5,206.52	7,172.78	18,066.91	15,010.30	21,384.77
Jordan	983.67	831	1,147.47	3,675.06	2,920.58	4,634.96
Kuwait	484.51	416.87	557.62	1,733.69	1,429.18	2,055.29
Lebanon	1,029.36	873.84	1,192.05	2,263.30	1,886.79	2,695.80
Libya	1,419.21	1,196.89	1,666.28	3,143.65	2,618.02	3,707.74
Morocco	11,786.05	10,042.92	13,600.20	17,882.33	15,008.41	21,147.80
Oman	440.74	369.51	516.9	1,438.23	1,153.75	1,739.23
Palestine	426.69	350.18	522.6	1,557.17	1,241.16	1,948.97
Qatar	175.24	150.94	200.31	1,249.27	1,048.16	1,462.15
Saudi Arabia	2,668.21	2,175.08	3,282.49	15,355.59	12,601.82	17,906.54
Sudan	8,027.19	6,750.56	9,409.15	19,021.30	15,326.63	23,351.60
Syrian Arab Republic	2,282.00	1,868.61	2,789.69	4,134.98	3,272.06	5,191.67
Tunisia	2,498.67	2,109.54	2,917.34	4,087.78	3,382.42	4,809.16
Türkiye	17,679.13	15,106.48	20,140.35	27,647.74	23,354.29	32,341.81
United Arab Emirates	619.08	536.3	707.66	4,077.51	3,357.01	4,774.41
Yemen	3,630.16	2,988.69	4,326.80	12,182.95	9,804.32	14,951.23

**Figure 4:**
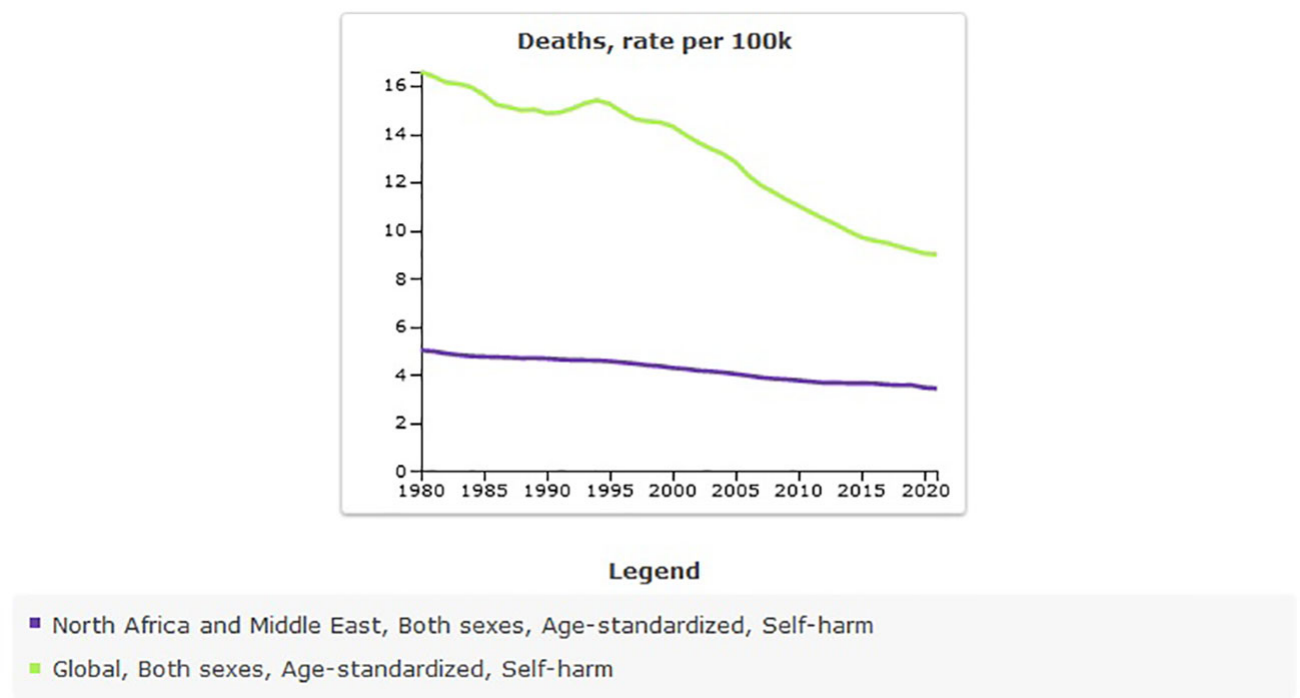
Trend in all-age counts incidence of self-harm in MENA, 1990–2021.

The all-age count estimates that the incidence of self-harm in MENA was 121,495 [95% UI 101,913 to 142,793] in 1990, and this rate increased to 237,245 [95% UI 194,870 to 284,896] in 2021. The percentage change from 1990 to 2021 showed a sharp increase of 95% [95% UI 83 to 111] (**Figure 4**).

### Disability-Adjusted Life Years of self-harm in MENA from 1990 to 2021

**Figure 5:**
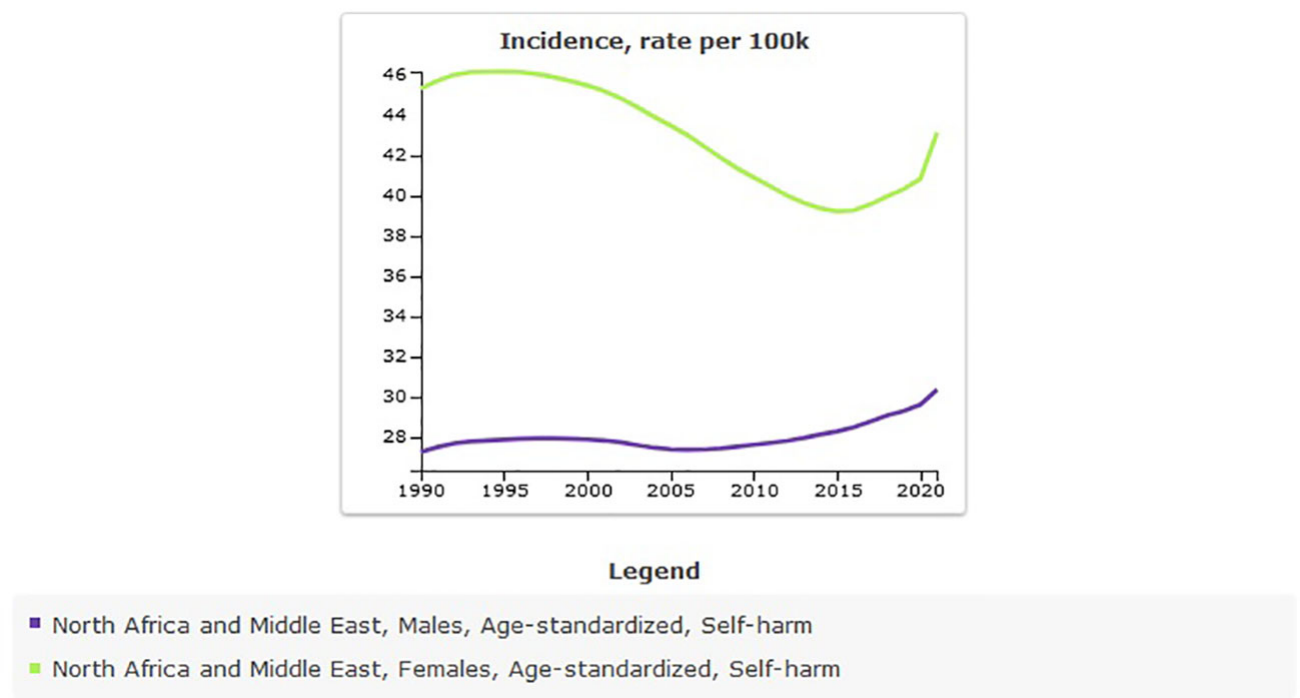
Trend in age-standardized DALYs of self-harm in MENA, 1990–2021.

The age-standardized DALYs rate of self-harm in MENA was 246.03 [95% UI 199.36 to 274.9] per 100,000 in 1990, and there was a decrease to 177.44 [95% UI 147.61 to 203.87] per 100,000 in 2021 (**Table 3**). The percentage change from 1990 to 2021 showed a negative trend of −0.28% [95% UI −0.37 to −0.06] (**Figure 5**).

**Table 3:** All-ages counts and age-standardized DALYs rate (Per 100,000) of self-harm in MENA, 1990–2021.

Location	1990			2021		
	Value	Lower	Upper	Value	Lower	Upper
Age-standardized DALYs Rate (Per 100,000)						
North Africa and Middle East	246.03	199.36	274.9	177.44	147.61	203.87
Afghanistan	309.85	224.4	456.28	249.15	186.92	372.64
Algeria	248.98	166.33	307.51	150.52	104.85	190.44
Bahrain	342.52	303.18	389.51	195	161.2	235.07
Egypt	81.01	63.12	92.04	60.42	49.36	72.75
Iran	350.18	287.04	382.31	226.63	202.91	250.46
Iraq	366.26	290.54	445.84	233.37	178.5	311.05
Jordan	153.29	120.02	180.74	58.31	47.22	72.12
Kuwait	82.61	76.18	90.22	89.93	75.98	108.05
Lebanon	92.32	50.16	120.15	47.79	39.72	57.6
Libya	236.3	162.95	300.54	259.25	171.36	341.21
Morocco	282.22	187.5	356.13	183.29	119.06	265.59
Oman	97.92	73.35	130.57	52.81	42.16	65.24
Palestine	57.36	42.43	75.65	48.17	39	56.51
Qatar	355.05	290.54	436.17	168.25	129.04	218.31
Saudi Arabia	433.87	316.2	558.66	321.88	242.38	418.83
Sudan	359.56	209.94	480.47	252.62	149.03	368.66
Syrian Arab Republic	92.33	70.01	114.63	46.39	35.73	61.23
Tunisia	188.83	131.66	255.84	148.67	101.52	209.82
Türkiye	221.23	154.28	303.57	185.79	137.11	224.5
United Arab Emirates	255.85	190.76	353.16	141.91	113.97	186.64
Yemen	275.99	131.98	397.4	221.61	106.35	333.59
All-ages counts estimates						
North Africa and Middle East	777,963.86	620,531.05	873,035.65	1,147,638.50	957,243.58	1,320,562.77
Afghanistan	24,885.81	17,826.90	37,555.51	62,940.69	45,463.07	102,634.93
Algeria	60,094.12	39,395.68	75,236.23	66,709.64	46,799.23	84,751.44
Bahrain	1,788.43	1,567.75	2,032.10	3,577.95	2,967.78	4,286.76
Egypt	41,470.39	32,207.95	48,087.82	61,595.03	49,813.55	74,578.75
Iran	188,787.36	148,375.22	206,949.05	203,257.54	183,865.89	225,136.26
Iraq	56,487.32	45,093.27	69,488.98	95,630.26	73,065.88	128,089.38
Jordan	5,209.86	3,964.08	6,227.24	7,590.38	6,131.49	9,386.95
Kuwait	1,501.65	1,379.65	1,644.49	4,903.51	4,133.08	5,859.56
Lebanon	2,649.40	1,446.08	3,452.94	2,860.92	2,378.27	3,431.60
Libya	9,243.76	6,305.07	11,773.53	20,542.27	13,643.68	27,012.70
Morocco	70,041.54	45,198.32	89,536.89	71,107.29	46,188.12	103,354.72
Oman	1,745.07	1,306.50	2,329.22	2,812.31	2,193.16	3,563.12
Palestine	976.85	732.32	1,277.67	2,424.22	1,942.15	2,859.39
Qatar	1,630.32	1,336.33	2,018.19	6,273.00	4,723.79	8,185.41
Saudi Arabia	61,138.35	44,805.17	79,658.40	155,463.77	113,368.89	206,734.33
Sudan	65,540.44	37,725.06	88,439.14	110,479.44	64,446.19	163,555.63
Syrian Arab Republic	10,151.66	7,680.66	12,613.15	6,874.12	5,306.08	9,126.81
Tunisia	15,403.48	10,643.53	20,796.99	18,264.59	12,327.58	25,762.43
Türkiye	126,836.34	87,336.86	176,140.41	163,725.32	121,424.92	198,256.36
United Arab Emirates	4,749.07	3,497.32	6,758.30	12,560.36	9,969.94	16,626.25
Yemen	27,207.06	11,557.43	40,190.52	66,975.47	30,251.68	102,680.75

The number of DALYs due to self-harm in MENA was 777,963 [95% UI 620,531 to 873,035] in 1990, and this rate increased to 1,147,638 [95% UI 957,243 to 1,320,562] by 2021. The percentage change from 1990 to 2021 showed a sharp increase of 48% [95% UI 29 to 92].

### Suicide mortality in MENA from 1990 to 2021

**Figure 6:**
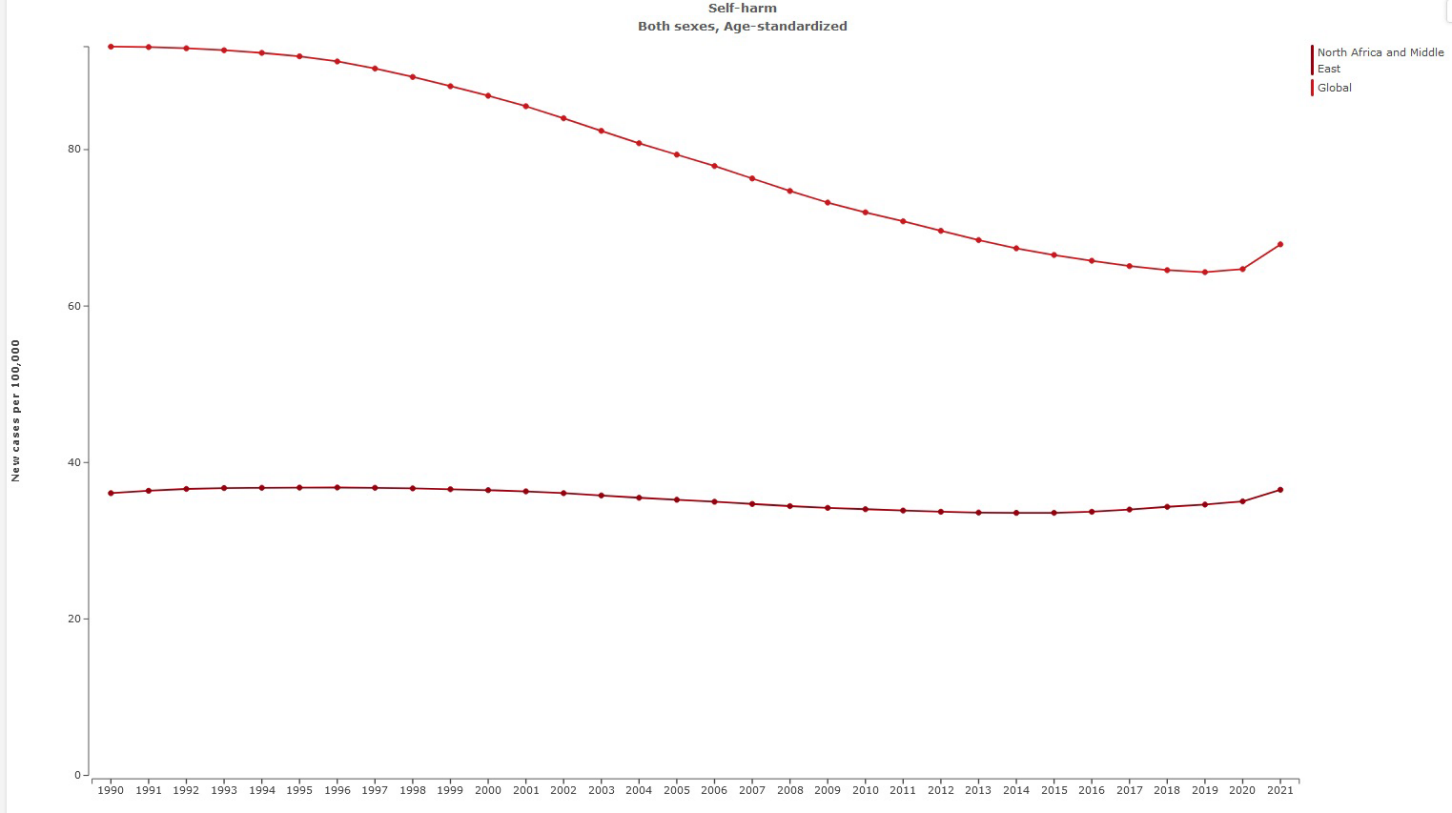
Trend in age-standardized suicide mortality rate in MENA, 1990–2021.

**Figure 7:**
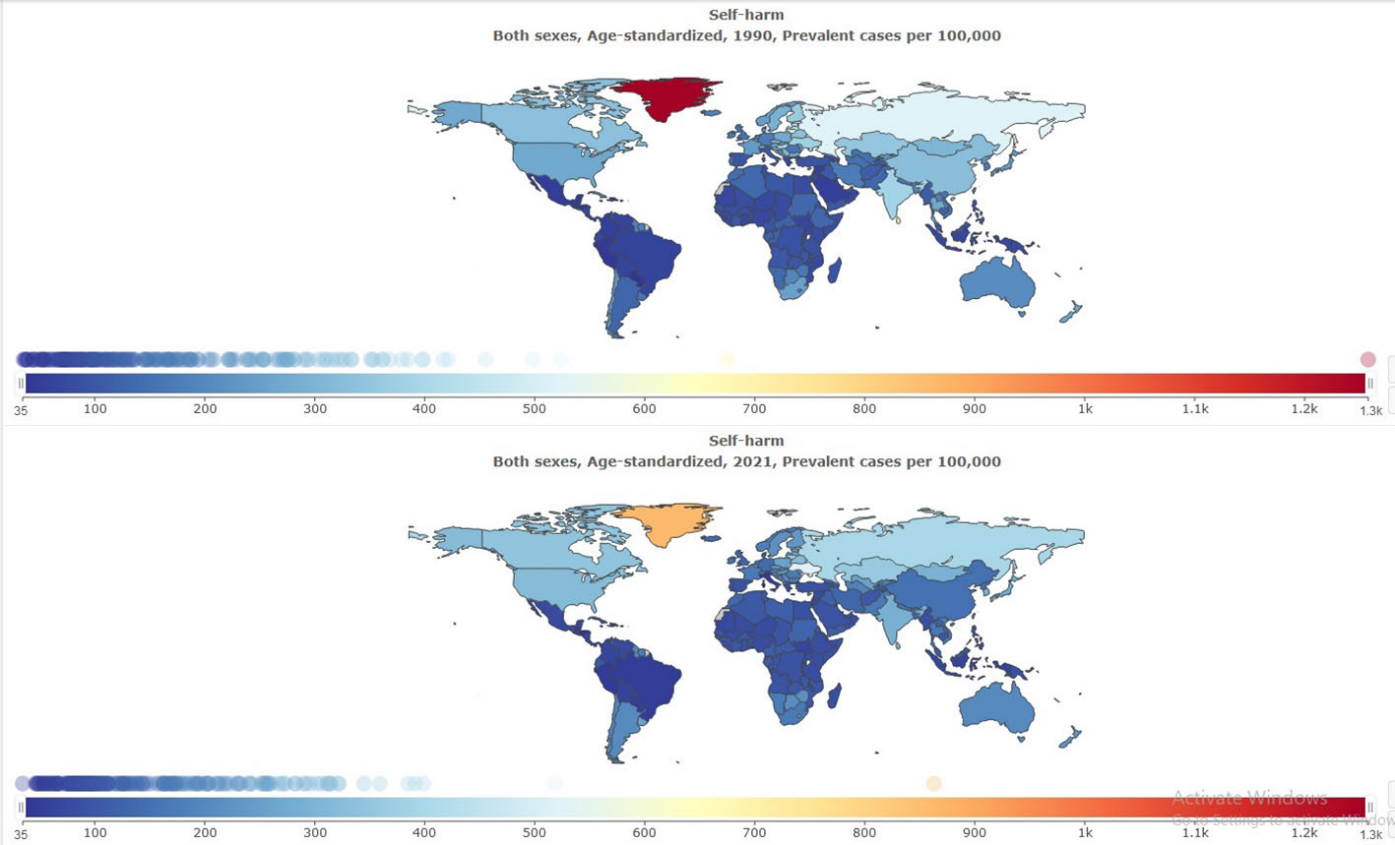
Age-standardized suicide mortality rate globally, 1990-2021.

The age-standardized rate of suicide mortality in MENA was 4.67 [95% UI 3.74 to 5.24] per 100,000 in 1990, and this rate decreased to 3.43 [95% UI 2.81 to 3.94] per 100,000 in 2021 (**Table 4**). The percentage change from 1990 to 2021 showed a negative trend of −27% [95% UI −36 to −4]. The global age-standardized rate of suicide mortality is 8.99 per 100,000 individuals in 2021. Compared with the global population, suicide mortality was less prevalent in MENA from 1990 to 2021 (**Figure 6**). The prevalence of suicide mortality in MENA countries was lower than in other countries (**Figure 7**).

**Table 4:** All-ages counts and age-standardized rate (Per 100,000) of suicide mortality in MENA, 1990–2021.

Location	1990			2021		
	Value	Lower	Upper	Value	Lower	Upper
Age-standardized suicide mortality (Per 100,000)						
North Africa and Middle East	4.67	3.74	5.24	3.43	2.81	3.94
Afghanistan	6.74	4.94	9.15	5.32	3.98	7.4
Algeria	4.54	3.01	5.57	2.85	1.96	3.63
Bahrain	7.29	6.52	8.24	3.93	3.28	4.8
Egypt	1.51	1.12	1.7	1.14	0.91	1.39
Iran (Islamic Republic of)	6.26	5.26	6.9	4.12	3.72	4.58
Iraq	7.37	5.86	8.86	4.89	3.68	6.46
Jordan	2.96	2.28	3.48	1.1	0.88	1.37
Kuwait	1.59	1.47	1.74	1.78	1.49	2.13
Lebanon	1.68	0.86	2.19	0.86	0.7	1.04
Libya	4.42	3.03	5.59	4.93	3.2	6.78
Morocco	5.17	3.41	6.54	3.59	2.3	5.04
Oman	1.91	1.4	2.58	1.02	0.8	1.28
Palestine	1.08	0.78	1.45	0.89	0.71	1.06
Qatar	7.74	6.3	9.49	3.41	2.6	4.48
Saudi Arabia	9.18	6.62	11.79	6.64	5.04	8.57
Sudan	6.61	3.83	8.79	4.68	2.78	6.77
Syrian Arab Republic	1.73	1.29	2.15	0.87	0.65	1.16
Tunisia	3.63	2.47	5.15	2.88	1.89	4.16
Türkiye	4.27	3	5.78	3.58	2.69	4.33
United Arab Emirates	5.27	3.91	7.17	2.76	2.21	3.61
Yemen	5.62	2.83	7.99	4.45	2.19	6.53
All-ages counts estimates						
North Africa and Middle East	13,332.59	10,628.81	14,969.02	21,195.57	17,412.70	24,366.77
Afghanistan	510.82	371.17	703.41	1,124.18	812.67	1,735.13
Algeria	982.55	639.13	1,224.09	1,221.94	845.11	1,565.66
Bahrain	31.77	28.14	36.07	65.46	54.29	79.42
Egypt	690.46	514.42	799.17	1,064.19	844.64	1,297.64
Iran (Islamic Republic of)	3,069.66	2,463.49	3,362.63	3,708.56	3,354.15	4,117.61
Iraq	1,009.43	798.17	1,235.49	1,819.85	1,371.48	2,435.05
Jordan	87.27	66.53	104.06	132.38	105.11	166.67
Kuwait	25.65	23.5	28.08	91.46	76	110.16
Lebanon	45.88	23.32	60.3	51.89	42.28	62.81
Libya	155.85	105.51	199.88	379.13	248.49	515.46
Morocco	1,183.60	769.64	1,511.53	1,367.01	874.92	1,937.08
Oman	30.11	22.12	40.71	49.07	37.69	62.68
Palestine	16.12	11.76	21.64	40.27	31.5	48.05
Qatar	28.91	23.57	35.92	113.51	84.04	148.41
Saudi Arabia	1,117.26	817.64	1,442.92	2,967.63	2,165.27	3,921.08
Sudan	1,096.78	625.39	1,481.35	1,857.14	1,077.14	2,736.46
Syrian Arab Republic	169.18	125.66	211.79	125.23	93.8	169.05
Tunisia	269.87	184.5	372.34	362.45	235.45	525.28
Türkiye	2,230.28	1,536.91	3,068.71	3,209.80	2,426.94	3,882.02
United Arab Emirates	83.29	60.78	118.74	228.59	177.92	307.89
Yemen	490.56	223.19	716.3	1,196.07	549.34	1,832.18

More than 21,000 suicide mortalities were estimated in MENA in 2021, which was estimate for 1990 was 13,332 [95% UI 10,628 to 14,969]. This shows that, despite the decrease the in age-standardized prevalence rate, the number of suicide deaths has increased.

### The sex-specific burden of self-harm and suicide mortality in MENA

Sex-specific prevalence of self-harm in MENA showed that the age-standardized prevalence rate (per 100,000) in 2021 was higher in females (112.57 [95% UI 95.65 to 134.77]) than in males (99.67 [95% UI 86.59 to 116.49]) (**Table 5**).

**Table 5:** Sex specific prevalence of self-harm in MENA, 1990–2021.

Location	Sex	1990			2021		
		Value	Lower	Upper	Value	Lower	Upper
Age-standardized prevalence Rate (Per 100,000)							
North Africa and Middle East	Males	95.63	83.89	110.96	99.67	86.59	116.49
Afghanistan	Males	67.85	59.56	78.74	71.93	62.35	83.33
Algeria	Males	100.06	85.69	118.41	88.7	76.54	105.15
Bahrain	Males	103.11	89.89	119.14	124.6	106.42	149.36
Egypt	Males	55.74	49.15	64.06	80.32	69.82	93.61
Iran (Islamic Republic of)	Males	136.66	116.78	162.01	137.62	116.74	162.53
Iraq	Males	101.13	89.28	116.73	118.6	103.09	137.12
Jordan	Males	78.69	69.42	90.53	67.17	58.8	77.89
Kuwait	Males	69.81	60.42	81.27	85.92	74.75	100.53
Lebanon	Males	81.96	70.91	96.32	105.49	90.67	124.38
Libya	Males	88.97	76.8	104.04	105.37	90.76	122.9
Morocco	Males	123.01	107.32	141.98	122.52	106.02	142.08
Oman	Males	80.43	70.09	93.78	80.36	68.59	96.17
Palestine	Males	80.84	70.31	94	81.13	70.31	95.39
Qatar	Males	100.15	87.97	116.18	97.1	84.43	113.41
Saudi Arabia	Males	52.94	46.67	60.47	99.41	85.87	114.35
Sudan	Males	98.28	85.17	115.22	112.68	97.09	132.65
Syrian Arab Republic	Males	62.22	54.73	72.13	70.86	61.68	82.58
Tunisia	Males	74.39	65.02	86.35	91.53	79.18	107.21
Türkiye	Males	114.15	99.83	130.11	80.74	70.92	93.7
United Arab Emirates	Males	96.48	84.78	112.07	105.73	91.48	122.94
Yemen	Males	75.89	66.58	86.71	95.11	82.85	110.62
All-ages counts estimates							
North Africa and Middle East	Males	115,055.41	99,459.44	135,137.30	302,534.19	261,114.27	356,524.46
Afghanistan	Males	2,323.95	2,047.90	2,676.30	6,645.52	5,746.66	7,816.53
Algeria	Males	8,550.55	7,243.75	10,283.70	19,230.63	16,549.88	22,839.61
Bahrain	Males	253.08	217.7	299.93	1,389.07	1,176.82	1,683.94
Egypt	Males	11,334.74	9,875.68	13,161.94	36,418.42	31,501.37	42,611.71
Iran (Islamic Republic of)	Males	25,980.61	21,921.03	31,313.86	64,913.99	54,867.53	76,963.05
Iraq	Males	5,794.81	5,046.07	6,780.73	20,673.39	17,830.47	24,171.28
Jordan	Males	909.67	784.62	1,058.58	3,941.25	3,416.27	4,617.59
Kuwait	Males	599.1	510.16	713.35	2,438.45	2,099.72	2,888.11
Lebanon	Males	997.43	860.25	1,172.77	3,063.87	2,620.00	3,644.77
Libya	Males	1,343.33	1,149.48	1,588.39	3,951.76	3,389.26	4,642.88
Morocco	Males	11,239.79	9,743.30	13,048.32	23,196.60	20,033.60	26,952.59
Oman	Males	706.31	602.63	843.58	2,373.74	1,978.93	2,920.03
Palestine	Males	465.34	397.87	550.16	1,586.35	1,355.86	1,881.70
Qatar	Males	278.03	237.15	329.97	2,299.57	1,954.46	2,754.60
Saudi Arabia	Males	3,369.31	2,914.77	3,913.57	23,196.17	19,808.24	27,251.07
Sudan	Males	6,355.42	5,491.06	7,490.40	17,626.49	15,124.83	20,761.13
Syrian Arab Republic	Males	2,449.77	2,124.84	2,865.44	4,884.30	4,285.46	5,632.28
Tunisia	Males	2,394.33	2,081.45	2,813.13	6,025.98	5,212.24	7,049.40
Türkiye	Males	25,831.83	22,509.35	29,768.34	37,601.30	33,002.38	43,689.79
United Arab Emirates	Males	1,039.20	885.61	1,247.40	10,088.48	8,571.49	11,892.68
Yemen	Males	2,775.87	2,428.80	3,198.96	10,706.70	9,277.27	12,673.68
Age-standardized prevalence Rate (Per 100,000)							
North Africa and Middle East	Females	128.83	109.52	152.86	112.57	95.65	134.77
Afghanistan	Females	151.7	131.25	175.82	133.86	116.17	155.33
Algeria	Females	169.7	143.41	202.27	118.89	100.8	142.87
Bahrain	Females	85.12	72.31	101.59	109.78	92.36	131.87
Egypt	Females	108.88	93.04	128.8	97.37	83.55	114.8
Iran (Islamic Republic of)	Females	212.79	176.25	258.02	124.82	104.03	151.66
Iraq	Females	117.53	100.72	138.8	118.31	101.18	141.68
Jordan	Females	85.92	74.81	99.85	88.03	74.88	104.63
Kuwait	Females	78.94	68.01	92.79	92.05	78.35	109.73
Lebanon	Females	120.85	103.19	144.65	109.92	92.99	132.87
Libya	Females	118.78	100.28	139.25	123.16	104.24	147.67
Morocco	Females	164.51	139.66	194.07	146.47	125.2	172.21
Oman	Females	63.04	54.85	73.64	86.7	73.97	102.37
Palestine	Females	62.09	54.29	72.33	86.78	74.12	103.4
Qatar	Females	110.24	95.16	129.56	96.17	82.27	113.05
Saudi Arabia	Females	54.39	47.13	63.95	88.44	75.31	104.27
Sudan	Females	162.84	137.95	194.53	140.64	118.65	169.01
Syrian Arab Republic	Females	58.02	49.95	68.09	85.9	73.48	101.68
Tunisia	Females	102.18	87.33	120.96	101.12	85.97	120.86
Türkiye	Females	77.53	67.24	90.26	99.13	84.14	119.34
United Arab Emirates	Females	99.07	87.57	113.2	105.69	91.58	124.17
Yemen	Females	124.11	107.25	145.24	127.85	109.17	151.32
All-ages counts estimates							
North Africa and Middle East	Females	152,249.20	128,889.25	182,579.38	318,975.58	269,387.72	383,923.59
Afghanistan	Females	5,585.52	4,852.08	6,482.66	12,212.50	10,445.08	14,348.00
Algeria	Females	14,696.79	12,383.45	17,732.75	25,444.52	21,454.19	30,681.62
Bahrain	Females	137.03	114.9	166.13	622.3	519.85	754.7
Egypt	Females	22,155.67	18,800.13	26,400.42	41,202.29	34,986.61	49,101.34
Iran (Islamic Republic of)	Females	39,854.26	32,912.27	49,170.48	57,867.77	48,020.96	70,468.16
Iraq	Females	6,727.47	5,695.31	8,019.78	19,883.63	16,844.31	24,003.92
Jordan	Females	923.69	789.27	1,092.72	4,384.17	3,700.27	5,279.68
Kuwait	Females	415.48	352.69	499.14	2,234.86	1,867.44	2,707.61
Lebanon	Females	1,597.96	1,359.35	1,922.69	3,472.72	2,933.94	4,196.58
Libya	Females	1,489.08	1,254.79	1,765.20	4,382.60	3,684.05	5,292.07
Morocco	Females	15,832.75	13,405.93	18,851.19	28,008.38	23,919.43	32,988.68
Oman	Females	303.66	260.63	362.23	1,335.63	1,123.94	1,597.11
Palestine	Females	400.15	344.91	470.26	1,695.51	1,426.04	2,039.79
Qatar	Females	112.87	95.28	136.35	781.1	650.5	941.34
Saudi Arabia	Females	2,378.73	2,020.80	2,855.31	13,812.42	11,610.60	16,546.25
Sudan	Females	10,984.61	9,223.03	13,171.21	22,448.50	18,761.31	27,341.78
Syrian Arab Republic	Females	2,242.39	1,913.83	2,679.00	6,373.21	5,456.16	7,563.77
Tunisia	Females	3,287.56	2,798.52	3,922.40	6,944.79	5,909.80	8,265.87
Türkiye	Females	17,870.73	15,340.99	20,987.87	47,479.15	40,439.47	56,975.47
United Arab Emirates	Females	392.38	336.85	463.43	2,969.09	2,534.81	3,520.18
Yemen	Females	4,777.13	4,105.67	5,651.56	15,122.93	12,774.45	18,124.23

The incidence of self-harm in MENA showed that the age-standardized incidence rate (per 100,000) in 2021 was higher in females (43.12 [95% UI 35.33to 52.24]) than in males (30.36 [95% UI 25.33 to 35.93]) (**Table 6**).

**Table 6:** Sex specific incidence of self-harm in MENA, 1990–2021.

Location	Sex	1990			2021		
		Value	Lower	Upper	Value	Lower	Upper
Age-standardized incidence Rate (Per 100,000)							
North Africa and Middle East	Males	27.27	23.36	31.05	30.36	25.33	35.93
Afghanistan	Males	18.37	15.69	20.98	19.93	16.42	23.81
Algeria	Males	27.91	23.48	32.58	26.65	21.87	32.34
Bahrain	Males	30.94	27.15	35.1	39.55	34.25	45.42
Egypt	Males	15.6	13.15	18.12	24.2	19.69	29.46
Iran (Islamic Republic of)	Males	39.72	32.38	48.02	42.9	33.59	53.18
Iraq	Males	29.66	25.33	34.31	37.29	31.47	43.11
Jordan	Males	23.2	20.28	26.06	20.97	17.8	24.75
Kuwait	Males	21.41	18.89	24.07	27.75	24.09	31.72
Lebanon	Males	24.05	20.34	27.87	33.41	27.51	40.18
Libya	Males	25.53	21.9	29.6	32.43	26.99	38.64
Morocco	Males	35.07	30.08	39.85	37.05	30.99	44.37
Oman	Males	23.73	20.57	27.05	25.28	21.12	30.25
Palestine	Males	23.33	20.31	26.47	24.72	21.08	28.97
Qatar	Males	30.37	26.09	34.31	31.48	26.67	36.99
Saudi Arabia	Males	15.12	12.99	17.44	31.94	26.73	37.17
Sudan	Males	27.37	23.08	31.8	32.65	26.75	39.17
Syrian Arab Republic	Males	17.77	15.64	20.04	21.7	18.38	25.54
Tunisia	Males	21.54	18.37	24.46	28.57	23.68	33.83
Türkiye	Males	32.46	28.62	36.12	25.27	21.82	29.16
United Arab Emirates	Males	28.25	24.59	31.72	32.62	27.47	38.28
Yemen	Males	21.68	18.35	25.11	27.54	23.04	32.72
All-ages counts estimates							
North Africa and Middle East	Males	46,055.30	38,935.11	53,408.96	103,006.04	85,574.22	121,722.06
Afghanistan	Males	764.33	632.85	898.65	3,050.07	2,432.26	3,756.16
Algeria	Males	3,626.37	3,011.40	4,306.53	5,947.20	4,874.85	7,187.17
Bahrain	Males	105.86	91.14	121.39	465.88	404.19	528.87
Egypt	Males	4,326.66	3,582.59	5,125.11	13,127.93	10,508.07	16,102.08
Iran (Islamic Republic of)	Males	10,863.93	8,683.10	13,557.19	19,891.51	15,639.20	24,678.36
Iraq	Males	2,586.13	2,169.17	3,036.84	8,333.66	6,927.53	9,795.16
Jordan	Males	439.66	374.28	508.12	1,521.23	1,274.99	1,810.19
Kuwait	Males	229.22	202.96	259.08	780.38	687.26	883.25
Lebanon	Males	349.17	291.16	416.42	990.21	813.57	1,190.97
Libya	Males	569.68	476.9	670.32	1,320.58	1,090.06	1,560.88
Morocco	Males	4,324.74	3,688.63	4,948.28	7,225.46	6,042.34	8,646.40
Oman	Males	279.24	237.75	321.21	780.76	643.36	934.82
Palestine	Males	219.67	185.11	259.36	677.44	569.14	817.42
Qatar	Males	112.52	96.38	128.96	867.36	734.43	1,005.03
Saudi Arabia	Males	1,380.11	1,165.60	1,617.39	8,849.34	7,352.84	10,251.07
Sudan	Males	2,525.86	2,108.08	2,963.71	7,368.57	5,924.11	9,058.95
Syrian Arab Republic	Males	1,072.28	913.87	1,245.96	1,490.06	1,238.40	1,804.55
Tunisia	Males	921.11	774.54	1,070.00	1,700.56	1,420.30	1,994.00
Türkiye	Males	9,764.26	8,450.14	11,025.57	11,197.87	9,735.73	12,811.35
United Arab Emirates	Males	389.67	339.13	444.67	2,817.86	2,313.93	3,324.45
Yemen	Males	1,179.63	966.1	1,414.85	4,506.05	3,673.58	5,482.65
Age-standardized incidence Rate (Per 100,000)							
North Africa and Middle East	Females	45.3	38.49	52.72	43.12	35.33	52.24
Afghanistan	Females	54.82	47.72	62.68	49.37	41.67	57.67
Algeria	Females	58.98	51.01	67.94	45.89	38.31	54.69
Bahrain	Females	30.47	25.27	35.6	45.06	37.41	53.39
Egypt	Females	36.84	31.76	42.07	37.25	30.56	44.76
Iran (Islamic Republic of)	Females	75.31	60.81	92.21	49.28	38.52	62
Iraq	Females	41.88	36.31	48.1	45.99	38.25	54.44
Jordan	Females	32.06	27.85	36.61	34.78	26.53	43.88
Kuwait	Females	30.91	25.78	36.77	37.99	29.07	48.33
Lebanon	Females	44.06	37.83	50.57	44.94	37.58	53.63
Libya	Females	42.72	36.88	48.98	48.12	39.87	57.81
Morocco	Females	56.78	48.75	65.6	55.31	46.32	65.43
Oman	Females	22.79	18.76	27.39	35.12	27.24	43.92
Palestine	Females	21.88	17.85	26.78	33.06	25.82	41.73
Qatar	Females	41.54	36.07	47.61	40.57	33.13	49.43
Saudi Arabia	Females	18.68	14.93	23.13	35.61	28.27	43.98
Sudan	Females	54.77	46.98	63.24	50.6	41.02	61.1
Syrian Arab Republic	Females	20.19	16.39	24.99	33.07	25.78	41.36
Tunisia	Females	36.45	31.39	42.09	40	32.43	48.21
Türkiye	Females	27.06	23	31.86	38.75	31.36	47.41
United Arab Emirates	Females	37.42	32.56	42.94	41.65	33.95	50.19
Yemen	Females	43.07	36.15	50.09	45.68	37.26	55.33
All-ages counts estimates							
North Africa and Middle East	Females	75,440.61	63,161.37	89,493.70	134,239.27	109,704.29	163,264.05
Afghanistan	Females	2,467.33	2,117.68	2,887.47	6,808.00	5,599.21	8,272.44
Algeria	Females	7,553.55	6,351.81	8,849.35	9,956.58	8,335.40	11,798.28
Bahrain	Females	70.68	58.55	84.31	278.07	230.24	328.18
Egypt	Females	10,115.57	8,543.74	11,774.91	18,964.26	15,303.74	22,977.30
Iran (Islamic Republic of)	Females	21,219.21	16,839.31	26,855.28	21,017.07	16,503.98	26,128.95
Iraq	Females	3,579.94	3,039.98	4,179.76	9,733.26	8,010.47	11,659.25
Jordan	Females	544.02	450.89	651.2	2,153.83	1,608.78	2,771.91
Kuwait	Females	255.29	210.94	308.08	953.32	741.58	1,185.27
Lebanon	Females	680.18	577.92	791.9	1,273.10	1,073.97	1,503.73
Libya	Females	849.53	715.19	1,007.80	1,823.07	1,506.24	2,178.93
Morocco	Females	7,461.31	6,304.40	8,714.20	10,656.87	8,920.42	12,582.24
Oman	Females	161.5	127.94	202.84	657.46	506.19	829.21
Palestine	Females	207.02	160.84	265.09	879.73	662.96	1,137.79
Qatar	Females	62.72	54.13	72.6	381.91	303.74	459.97
Saudi Arabia	Females	1,288.10	986.02	1,653.58	6,506.26	5,131.79	7,818.94
Sudan	Females	5,501.33	4,622.98	6,499.25	11,652.73	9,308.09	14,379.60
Syrian Arab Republic	Females	1,209.72	940.27	1,564.50	2,644.92	1,999.37	3,381.75
Tunisia	Females	1,577.56	1,330.31	1,846.99	2,387.22	1,955.51	2,837.37
Türkiye	Females	7,914.86	6,554.23	9,576.53	16,449.87	13,398.54	20,057.14
United Arab Emirates	Females	229.41	195.14	267.54	1,259.65	1,015.77	1,505.50
Yemen	Females	2,450.53	2,024.05	2,916.47	7,676.90	6,126.53	9,461.56

**Figure 8:**
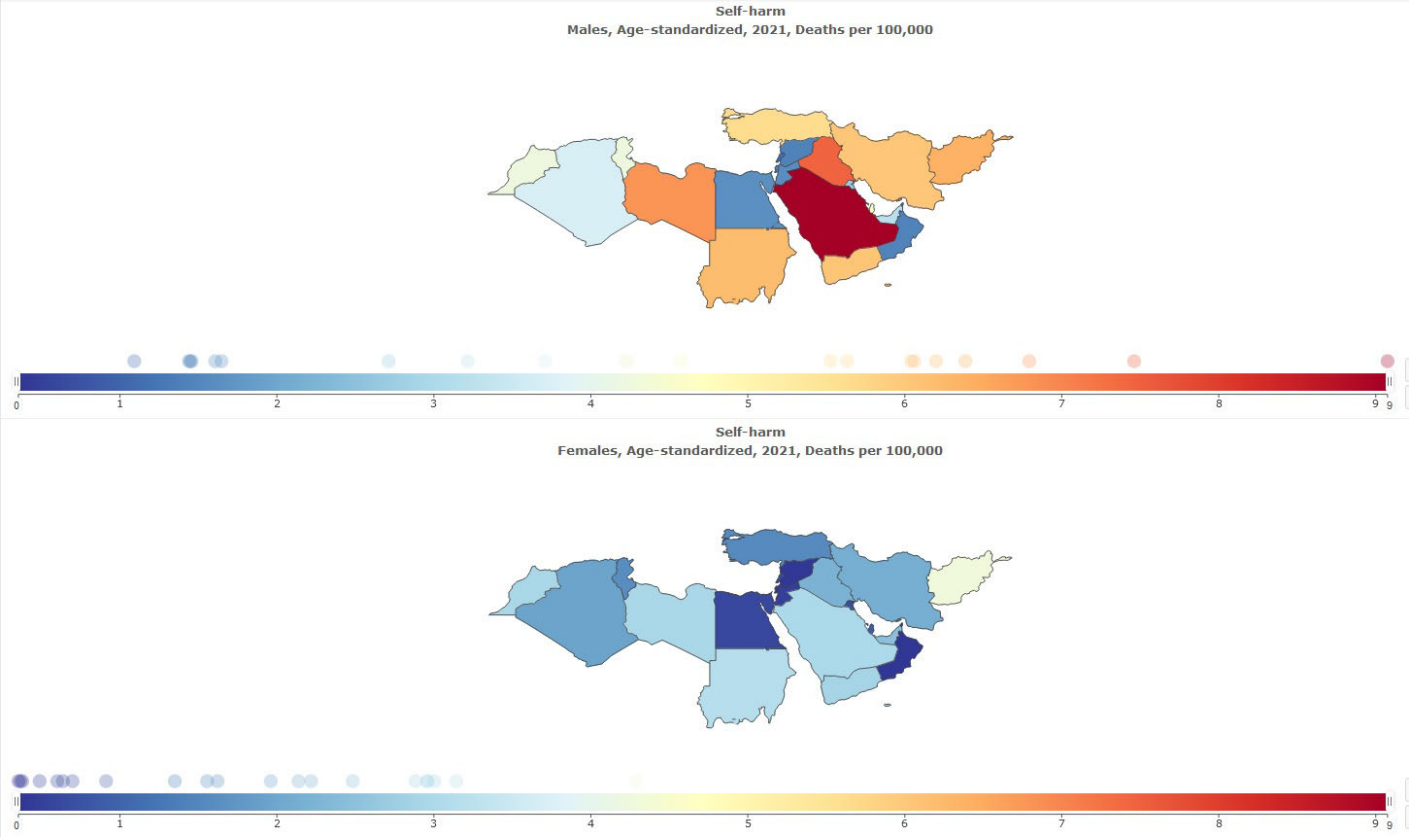
Sex specific age-standardized incidence rate of self-harm in MENA, 1990-2021.

The trend of incidence of self-harm in males and females from 1990 to 2021 showed that the trend in females decreased, but it increased in males (**Figure 8**). DALYs of self-harm rate per 100,000 in 2021 were higher in males (242.52 [95% UI 200.7 to 278.99]), than in females (106.64 [95% UI 85.25 to 128.14]). The trend of DALYs caused by self-harm in both sexes showed a decreasing trend compared with 1990 (**Table 7**).

**Table 7:** Sex specific DALYs of self-harm in MENA, 1990–2021.

Location	Sex	1990			2021		
		Value	Lower	Upper	Value	Lower	Upper
Age-standardized DALYs Rate (Per 100,000)							
North Africa and Middle East	Males	302.75	246.29	345.11	242.52	200.7	278.99
Afghanistan	Males	376.99	243.72	545.72	293.27	215.31	419.72
Algeria	Males	282.97	214.52	348.94	190.73	135.41	245.48
Bahrain	Males	434.98	374.36	512.67	263.11	214.52	319.53
Egypt	Males	106.13	83.32	126.32	84.85	68	103.16
Iran (Islamic Republic of)	Males	394.97	339.53	445.59	325.29	277.26	357.39
Iraq	Males	512.3	392.76	631.43	339.32	253.01	458.71
Jordan	Males	183.05	145.83	219.12	82.5	66.11	102.3
Kuwait	Males	95.54	85.89	106.95	134.91	111.58	164.47
Lebanon	Males	98.72	53.37	131.29	57.19	47.47	69.52
Libya	Males	280.02	193.03	363.69	350.13	241.08	457.11
Morocco	Males	280.02	211.81	360.62	205.41	142.49	313.27
Oman	Males	124.81	90.98	172.14	70.5	54.45	88.29
Palestine	Males	83.43	61.02	112.53	72.99	55.38	86.49
Qatar	Males	440.07	354.76	554.14	221.78	167.47	290.99
Saudi Arabia	Males	517.06	378.27	688.77	434.4	323.9	586.06
Sudan	Males	385.18	253.01	522.73	321.68	197.64	457.61
Syrian Arab Republic	Males	130.68	98.75	167.23	73.6	54.87	98.43
Tunisia	Males	220.23	166.39	336.62	211.41	141.28	307.61
Türkiye	Males	341.25	235.18	445.16	283.3	212.27	343.89
United Arab Emirates	Males	287.44	205.92	420.74	178.65	139.08	248.89
Yemen	Males	341.95	187.85	493.24	288.39	151.34	434.33
All-ages counts estimates							
North Africa and Middle East	Males	478,630.60	389,396.59	546,563.14	817,712.72	677,563.34	941,813.38
Afghanistan	Males	13,719.84	9,083.67	19,986.06	37,226.62	26,865.39	54,632.73
Algeria	Males	34,001.22	25,211.82	42,555.66	42,849.37	30,381.18	55,206.63
Bahrain	Males	1,349.88	1,154.89	1,610.36	3,140.52	2,585.16	3,807.87
Egypt	Males	27,614.10	21,728.33	33,419.30	44,919.14	35,755.92	54,522.88
Iran (Islamic Republic of)	Males	105,109.67	88,800.06	118,654.48	150,642.85	129,386.56	165,530.23
Iraq	Males	39,289.40	30,621.57	49,225.45	71,402.32	52,565.55	97,663.20
Jordan	Males	3,209.08	2,512.14	3,911.12	5,880.51	4,673.51	7,359.85
Kuwait	Males	1,027.57	920.95	1,151.05	3,903.84	3,197.45	4,742.81
Lebanon	Males	1,338.15	730.27	1,810.55	1,727.38	1,427.41	2,106.62
Libya	Males	5,803.72	3,955.71	7,708.27	14,339.55	9,931.11	18,681.82
Morocco	Males	32,989.46	24,889.81	43,534.61	39,905.46	27,645.22	61,069.07
Oman	Males	1,409.20	1,025.29	1,948.61	2,402.35	1,840.78	3,071.48
Palestine	Males	691.04	499.78	935.19	1,872.35	1,415.20	2,244.01
Qatar	Males	1,395.26	1,119.95	1,775.76	5,863.43	4,401.58	7,717.36
Saudi Arabia	Males	42,138.02	30,493.61	56,042.06	125,162.98	88,930.30	172,548.80
Sudan	Males	32,579.45	21,514.94	44,818.03	68,362.29	41,007.18	98,811.41
Syrian Arab Republic	Males	7,221.81	5,363.71	9,432.67	4,959.15	3,684.60	6,771.38
Tunisia	Males	8,886.06	6,632.13	13,595.55	12,828.29	8,552.82	18,754.00
Türkiye	Males	98,639.19	68,224.01	129,932.04	126,613.82	94,875.49	153,671.33
United Arab Emirates	Males	3,695.62	2,671.69	5,599.87	10,530.57	8,262.72	14,285.00
Yemen	Males	16,261.05	8,155.58	23,951.62	42,417.24	20,972.65	64,665.56
Age-standardized DALYs Rate (Per 100,000)							
North Africa and Middle East	Females	186.34	126.28	212.64	106.64	85.25	128.14
Afghanistan	Females	256.35	184.77	425.17	204.75	144.38	354.49
Algeria	Females	214.76	102.43	291.02	109.44	64.2	145.98
Bahrain	Females	203.43	155.02	240.33	71.64	57.77	102.83
Egypt	Females	54.9	35.97	67.31	34.44	26.65	43.49
Iran (Islamic Republic of)	Females	303.11	202.92	343.37	124.44	108.73	155.18
Iraq	Females	212.3	152.16	265.78	119.43	89.16	170.84
Jordan	Females	120.14	75.2	155.9	29.01	22.55	39.19
Kuwait	Females	63.89	58.28	72.54	39.93	34.72	46.25
Lebanon	Females	86.96	43.47	120.59	38.67	29.64	49.94
Libya	Females	182.92	95.51	245.58	162.83	91.95	246.02
Morocco	Females	284.61	133.3	380.37	161.29	89.71	250.36
Oman	Females	51.71	33.18	69.14	22.94	17.87	29.41
Palestine	Females	32.9	23.98	45.57	22.72	18.4	29.42
Qatar	Females	174.11	122.8	221.46	46.17	34.7	66.07
Saudi Arabia	Females	304.46	195.54	399.87	155	111.84	215.98
Sudan	Females	333.76	132.31	501.24	184.99	84.37	306.45
Syrian Arab Republic	Females	52.42	34.63	66.92	24.45	18.38	35.4
Tunisia	Females	157.33	82.13	218.84	88.39	54.1	125.23
Türkiye	Females	99.03	60.7	191.88	86.74	60.01	109.94
United Arab Emirates	Females	186.33	135.43	246.55	95.11	72.86	137.02
Yemen	Females	210.12	65.15	331.61	155.87	60.35	238.86
All-ages counts estimates							
North Africa and Middle East	Females	299,333.25	201,107.98	342,548.29	329,925.78	263,651.16	396,673.90
Afghanistan	Females	11,165.97	7,820.10	19,123.92	25,714.07	17,573.87	52,174.13
Algeria	Females	26,092.90	11,959.03	35,662.48	23,860.27	14,016.64	31,950.35
Bahrain	Females	438.54	333.95	521.58	437.43	353.2	626.16
Egypt	Females	13,856.28	8,561.90	17,291.89	16,675.89	12,856.00	21,369.37
Iran (Islamic Republic of)	Females	83,677.70	53,304.15	95,584.67	52,614.69	45,850.79	66,073.28
Iraq	Females	17,197.92	12,211.37	21,938.07	24,227.94	18,021.65	34,927.77
Jordan	Females	2,000.77	1,246.68	2,656.76	1,709.87	1,315.35	2,334.15
Kuwait	Females	474.08	427.95	560.39	999.68	864.63	1,152.94
Lebanon	Females	1,311.25	651.06	1,812.65	1,133.54	875	1,471.12
Libya	Females	3,440.04	1,766.83	4,635.21	6,202.71	3,503.93	9,257.47
Morocco	Females	37,052.08	16,786.58	49,748.15	31,201.83	17,338.91	48,426.09
Oman	Females	335.88	208.89	448.5	409.96	315.04	529.5
Palestine	Females	285.82	204.56	396.88	551.86	439.82	724.43
Qatar	Females	235.06	165.24	300.43	409.57	300.1	610.62
Saudi Arabia	Females	19,000.33	11,740.29	25,207.64	30,300.80	21,675.04	41,521.78
Sudan	Females	32,960.98	12,541.19	49,104.75	42,117.16	18,696.05	71,008.30
Syrian Arab Republic	Females	2,929.85	1,953.85	3,781.58	1,914.97	1,432.63	2,778.14
Tunisia	Females	6,517.42	3,339.71	9,024.59	5,436.30	3,362.17	7,641.40
Türkiye	Females	28,197.15	16,880.92	55,953.71	37,111.50	25,959.59	46,687.57
United Arab Emirates	Females	1,053.45	754.42	1,425.32	2,029.79	1,554.58	2,890.45
Yemen	Females	10,946.01	3,066.25	17,863.75	24,558.23	8,851.05	38,673.30

**Figure 9:**
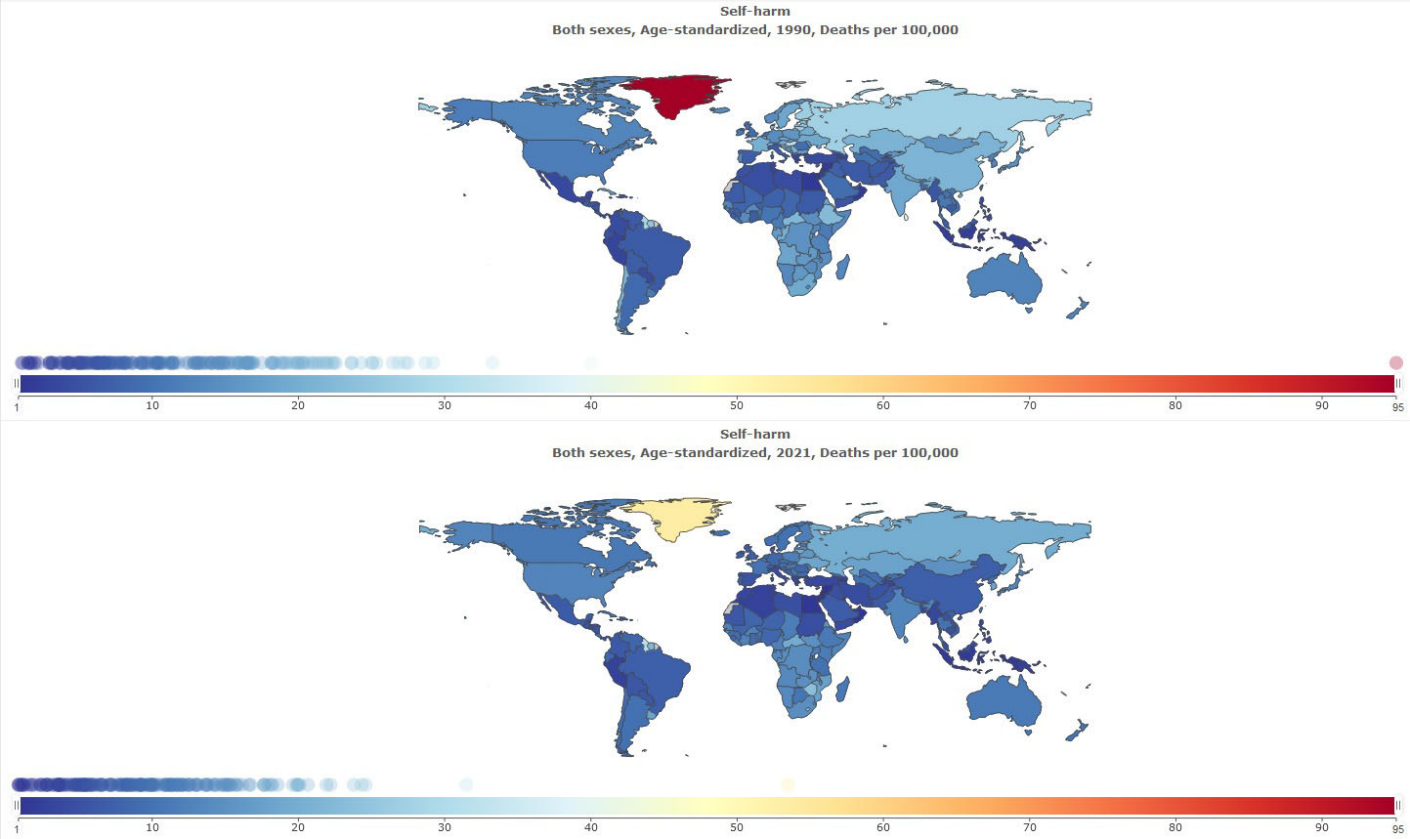
Sex specific age-standardized suicide mortality rate in MENA, stratified by country, 1990-2021.

Suicide mortality was more common in males than in females; the age-standardized prevalence rate (per 100,000) of suicide mortality in 2021 was 4.83 [95% UI 3.98 to 5.56] in males and 1.92 [95% UI 1.47 to 2.30] in females (**Table 8**, **Figure 9**).

**Table 8:** Sex specific suicide mortality in MENA, 1990–2021.

Location	Sex	1990			2021		
		Value	Lower	Upper	Value	Lower	Upper
Age-standardized prevalence Rate (Per 100,000)							
North Africa and Middle East	Males	6.02	4.92	6.83	4.83	3.98	5.56
Afghanistan	Males	8.36	5.6	11.58	6.39	4.69	8.86
Algeria	Males	5.38	4.16	6.57	3.71	2.59	4.78
Bahrain	Males	9.85	8.48	11.44	5.53	4.57	6.73
Egypt	Males	2.09	1.61	2.44	1.65	1.3	2.01
Iran (Islamic Republic of)	Males	7.42	6.35	8.39	6.04	5.14	6.67
Iraq	Males	10.82	8.37	13.19	7.46	5.47	9.88
Jordan	Males	3.64	2.88	4.32	1.61	1.28	1.97
Kuwait	Males	1.87	1.67	2.07	2.71	2.22	3.29
Lebanon	Males	1.92	1	2.57	1.09	0.89	1.34
Libya	Males	5.39	3.75	6.88	6.79	4.61	9.07
Morocco	Males	5.44	4.06	7	4.23	2.88	6.29
Oman	Males	2.53	1.83	3.49	1.46	1.12	1.83
Palestine	Males	1.67	1.19	2.24	1.45	1.08	1.72
Qatar	Males	9.87	7.93	12.28	4.57	3.47	6.05
Saudi Arabia	Males	11.41	8.33	15.13	9.08	6.73	11.82
Sudan	Males	7.55	4.83	10.22	6.2	3.81	8.89
Syrian Arab Republic	Males	2.52	1.88	3.21	1.44	1.05	1.96
Tunisia	Males	4.41	3.32	6.89	4.22	2.74	6.32
Türkiye	Males	6.73	4.65	8.71	5.63	4.22	6.83
United Arab Emirates	Males	5.93	4.28	8.67	3.22	2.53	4.42
Yemen	Males	7.3	4.31	10.17	6.06	3.3	8.9
All-ages counts estimates							
North Africa and Middle East	Males	8,514.15	6,925.22	9,709.15	15,485.87	12,846.80	17,904.47
Afghanistan	Males	294.04	199.01	409.46	664.42	479.24	969.98
Algeria	Males	573.6	431.28	712.25	808.5	567.56	1,035.65
Bahrain	Males	24.51	21.07	28.98	57.93	47.95	70.2
Egypt	Males	487.68	381.8	587.18	808.27	636.06	992.25
Iran (Islamic Republic of)	Males	1,779.40	1,522.33	2,017.04	2,802.33	2,398.13	3,090.06
Iraq	Males	726.93	560.79	896.8	1,399.53	1,038.63	1,890.04
Jordan	Males	54.95	42.97	66.6	105.85	84.18	131.83
Kuwait	Males	17.95	16.02	20.01	74.95	60.98	91.36
Lebanon	Males	24.67	12.79	33.26	32.7	26.67	40.06
Libya	Males	100.58	68.77	131.34	269.44	184.23	355.59
Morocco	Males	586.32	438.11	764.95	800.47	541.41	1,198.81
Oman	Males	24.71	17.77	34.16	43.02	32.75	55.64
Palestine	Males	11.74	8.39	16.04	32.42	24.09	39.02
Qatar	Males	24.97	19.93	31.94	106.75	79.35	140.56
Saudi Arabia	Males	794.34	574.66	1,058.87	2,411.92	1,734.66	3,298.88
Sudan	Males	578.42	377.65	789.45	1,193.00	717.81	1,706.73
Syrian Arab Republic	Males	123.63	91.19	160.74	96.88	70.46	134.23
Tunisia	Males	160.85	120.02	249.55	259.85	168.5	390.25
Türkiye	Males	1,749.42	1,202.73	2,295.31	2,523.86	1,892.59	3,076.91
United Arab Emirates	Males	65.77	47.55	99.99	193.61	147.9	266.68
Yemen	Males	305	163.11	442.51	785.72	400.57	1,191.67
Age-standardized prevalence Rate (Per 100,000)							
North Africa and Middle East	Females	3.26	2.21	3.75	1.92	1.47	2.3
Afghanistan	Females	5.32	3.92	7.52	4.29	3.07	6.44
Algeria	Females	3.7	1.67	5.03	1.96	1.1	2.61
Bahrain	Females	3.93	3.05	4.64	1.35	1.09	2.03
Egypt	Females	0.9	0.56	1.11	0.6	0.45	0.78
Iran (Islamic Republic of)	Females	5.04	3.47	5.71	2.14	1.85	2.74
Iraq	Females	3.8	2.64	4.73	2.22	1.61	3.16
Jordan	Females	2.2	1.31	2.86	0.49	0.36	0.68
Kuwait	Females	1.19	1.09	1.32	0.7	0.6	0.81
Lebanon	Females	1.46	0.66	2.06	0.64	0.47	0.84
Libya	Females	3.24	1.63	4.34	2.96	1.58	4.64
Morocco	Females	4.91	2.16	6.59	2.96	1.52	4.54
Oman	Females	0.92	0.57	1.29	0.37	0.27	0.49
Palestine	Females	0.55	0.39	0.79	0.35	0.28	0.45
Qatar	Females	3.53	2.48	4.42	0.91	0.68	1.35
Saudi Arabia	Females	5.86	3.68	7.67	3	2.17	4.18
Sudan	Females	5.65	2.08	8.41	3.14	1.37	5.29
Syrian Arab Republic	Females	0.89	0.56	1.15	0.38	0.27	0.57
Tunisia	Females	2.86	1.38	4.22	1.62	0.92	2.4
Türkiye	Females	1.84	1.14	3.48	1.56	1.05	1.99
United Arab Emirates	Females	3.82	2.76	5.01	2.48	1.87	3.58
Yemen	Females	3.98	1.2	6.26	2.88	1.07	4.44
All-ages counts estimates							
North Africa and Middle East	Females	4,818.45	3,196.71	5,541.97	5,709.70	4,370.65	6,872.70
Afghanistan	Females	216.79	156.72	323.66	459.76	314.56	843.8
Algeria	Females	408.94	181.7	562.95	413.44	235.83	558.18
Bahrain	Females	7.26	5.54	8.68	7.52	5.95	11.21
Egypt	Females	202.78	112.77	256.59	255.92	189.26	337
Iran (Islamic Republic of)	Females	1,290.26	820.94	1,468.85	906.23	779.55	1,182.62
Iraq	Females	282.5	199.42	357.67	420.32	305.22	607.21
Jordan	Females	32.32	19.55	42.65	26.53	19.63	37.51
Kuwait	Females	7.71	6.95	9.05	16.51	14.28	19.1
Lebanon	Females	21.21	9.45	30.08	19.18	14.4	25.37
Libya	Females	55.28	27.7	74.97	109.69	59.08	167.81
Morocco	Females	597.27	262.47	809.3	566.54	291.33	872.93
Oman	Females	5.4	3.29	7.42	6.05	4.4	8.19
Palestine	Females	4.38	2.99	6.28	7.86	6.24	10.37
Qatar	Females	3.94	2.78	5.07	6.76	4.81	10.35
Saudi Arabia	Females	322.91	201.41	426.67	555.71	395.19	765.25
Sudan	Females	518.36	189.01	784.06	664.14	276.11	1,136.47
Syrian Arab Republic	Females	45.55	29	59.87	28.35	20.1	42.86
Tunisia	Females	109.01	53.86	154.95	102.59	58.23	153.52
Türkiye	Females	480.86	286.79	951.21	685.94	468.43	878.16
United Arab Emirates	Females	17.52	12.44	23.96	34.99	26.03	51.21
Yemen	Females	185.56	49.83	301.68	410.34	140.19	649.39

## Discussion

This study deals with self-harm and suicide mortality in MENA from 1990 to 2021 based on sex and country. Based on this, the prevalence, incidence, DALYs, and suicide mortality in 21 countries according to sex were presented and compared with the global population.

This level of prevalence of self-harm compared to 1990 showed a decreasing trend, and it has also been shown that the estimated prevalence of self-harm is higher in females than in males. This decrease in self-harm over the last three decades may involve different mechanisms. The first point that is evident in MENA is that it especially affects mental health. Sociodemographic and religious commonalities are some of the factors that especially affect the tendency to self-harm and suicide mortality. In addition, the study shows that religiosity is protective against suicide (25–27). The dominant religion of the MENA countries is Islam, and most of the population in these countries are Muslims; according to Islamic teachings, self-harm is forbidden, and suicide is considered unforgivable from God’s point of view and prohibited by Islam (28, 29).

Previous studies have also shown a downward trend in suicide rates in this region (30). Similar to previous studies, this study showed that countries with Muslim populations have a lower prevalence of suicide (31, 32). Being religious is a protective factor against self-harm and suicide, even though this region has been involved in conflict, war, displacement, migration, and poverty for decades. In the past decades, this super region has witnessed an improvement in morbidity and mortality due to improvements in health care, health education, socioeconomic developments (11), and mental health infrastructure (33, 34). The stigma surrounding suicide can influence reported estimates, as many families choose to conceal deaths resulting from suicide due to the associated shame. The tendency to hide such cases can distort the accuracy of suicide-related statistics in the region.

However, the results clearly indicate an increase in the number of self-harm incidents and suicide-related deaths. Over the past three decades, the global population, as well as that of this region, has grown substantially (35), which requires an increase in health infrastructure and access to healthcare, especially mental healthcare. However, health systems in this region have faced challenges (34), which have affected the health performance of countries, and conflicts and wars have also increased the burden on the health system. As a result of conflicts and wars, migration also increases and people are displaced from their places of residence. As the literature also shows, there is an increase in mental disorders as a result of migration and displacement (15, 36). Accordingly, the increase in the number of self-harm and suicide deaths does not seem unusual.

Another finding of this study was the sex differences in the rate of self-harm and suicide mortality, which showed that self-harm is more common in females than in males, but the prevalence of suicide mortality is higher in males. The findings obtained from previous studies also confirm the higher prevalence of self-harm in females than in males; this sex difference has been shown in adolescents and adults (37, 38). Suicidal mortality has also been shown to be more common in males than females (39, 40). Mechanisms have been identified for the higher prevalence of self-harm in females (37). Individual, interpersonal, and sociocultural factors are associated with high levels of depression and distress in females (41). The tendency toward rumination also has an important relationship with self-harm (42). Depression is one of the main causes of suicide. Depression is more common in females, but suicide mortality is more common in males, and there is a paradox (39). In explaining these differences, three factors should be considered “methods used by suicide attempters, lethality of the suicidal act, and intent to die” (43). In females, the use of pills for suicide is more common, and in males, the use of firearms or hanging oneself is more common (44–47). The degree of suicide attempt among females is less lethal (46). There is a difference between males and females in the intention to die (48, 49); in men compared to females, this intention to die is more serious (43). In addition, some biological and cultural factors have been proposed to explain gender differences in suicidal behaviors, including glutaminergic activity (50), sleep time (51), spirituality, and coping skills (52). Therefore, the risk factors for suicidal behavior in women and men are different (53), and these should be considered in explanations.

Not all countries in the region had similar rates of self-harm and suicide mortality and there was a disparity. These differences can be influenced by many factors, some of which can be attributed to the performance of the health systems in these countries. In addition, there are cultural differences, as suicide has high stigma in some countries, which may have affected the estimates obtained. At the political level, some countries in this region have been involved in conflicts and long wars, which have affected the performance of the mental health care system. There is also inequality in economic conditions, such that some countries have a high level of welfare, and some other countries have a high level of poverty and economic and social problems. Therefore, any of these factors can affect differences in the rates of self-harm and suicide in these countries.

## Limitations

This was a comprehensive effort to examine the burden of self-harm and suicide mortality in MENA, covering more than three decades, assessing sex differences, and reporting decompositions in 21 countries. This research has limitations according to the Global Burden of Disease study, including the quality and collection of primary data and inconsistency of data availability. The challenges of measuring suicidal behaviors in conflict settings and the impact of missing or biased data should be considered. In addition to the methodological limitations that exist in GBD, some cultural limitations can also affect the estimation of the results in this region. For example, the stigmatization effects of self-harm and suicidal behaviors traditionally exist in this area, and, as a result, the estimates obtained may differ from the true extent. Most countries in this region have predominantly Muslim populations, and in Islam, self-harm and suicide are considered sins. These religious teachings can affect the estimates obtained because families may hide suicide. Raw data are not available for some countries or the data are not up-to-date, and these are extracted and estimated from other countries based on specific methodologies. This uncertainty, as noted in a previous study (Yan et al., 2024), may have affected the estimates.

## Conclusion

The findings of this study showed a trend of reducing self-harm and suicide mortality in MENA in recent decades. Self-harm was more common in females than males, but suicide mortality was more common in males. Despite the decrease in the rates of suicide mortality and self-harm, the rates of suicide mortality and self-harm have increased. Therefore, in this context, there is a need to provide structures related to mental health care, screening, prevention, and treatment in a more comprehensive manner, as well as to accurately identify risk factors. Another suggestion involves incorporating mental healthcare into the primary healthcare system. Additionally, providing structured education within families and schools can play a crucial role in enhancing mental health literacy, ultimately helping alleviate the impact of mental disorders.

## Data Availability

The data sources of this study were taken from GBD 2021, which is publicly available here: https://vizhub.healthdata.org/gbd-results/ https://vizhub.healthdata.org/gbd-compare/.
